# Systems biology analyses reveal enhanced chronic morphine distortion of gut-brain interrelationships in simian human immunodeficiency virus infected rhesus macaques

**DOI:** 10.3389/fnins.2022.1001544

**Published:** 2022-10-13

**Authors:** Omalla A. Olwenyi, Samuel D. Johnson, Mehdi Bidokhti, Vandana Thakur, Kabita Pandey, Michellie Thurman, Arpan Acharya, Srijayaprakash Uppada, Shannon Callen, Luis Giavedoni, Udaykumar Ranga, Shilpa J. Buch, Siddappa N. Byrareddy

**Affiliations:** ^1^Department of Pharmacology and Experimental Neuroscience, University of Nebraska Medical Center, Omaha, NE, United States; ^2^Department of Pathology and Microbiology, University of Nebraska Medical Center, Omaha, NE, United States; ^3^Department of Biology, Trinity University, San Antonio, TX, United States; ^4^Molecular Biology and Genetics Unit, Jawaharlal Nehru Centre for Advanced Scientific Research, Bangalore, India; ^5^Department of Genetics, Cell Biology and Anatomy, University of Nebraska Medical Center, Omaha, NE, United States; ^6^Department of Biochemistry and Molecular Biology, University of Nebraska Medical Center, Omaha, NE, United States

**Keywords:** morphine, gut-brain axis, microbiome, microglia, SHIV, neurogenesis, CNS, viral reservoirs

## Abstract

**Background:**

Commonly used opioids, such as morphine have been implicated in augmented SIV/HIV persistence within the central nervous system (CNS). However, the extent of myeloid cell polarization and viral persistence in different brain regions remains unclear. Additionally, the additive effects of morphine on SIV/HIV dysregulation of gut-brain crosstalk remain underexplored. Therefore, studies focused on understanding how drugs of abuse such as morphine affect immune dynamics, viral persistence and gut-brain interrelationships are warranted.

**Materials and methods:**

For a total of 9 weeks, rhesus macaques were ramped-up, and twice daily injections of either morphine (*n* = 4) or saline (*n* = 4) administered. This was later followed with infection with SHIVAD8EO variants. At necropsy, mononuclear cells were isolated from diverse brain [frontal lobe, cerebellum, medulla, putamen, hippocampus (HIP) and subventricular zone (SVZ)] and gut [lamina propria (LP) and muscularis (MUSC) of ascending colon, duodenum, and ileum] regions. Multiparametric flow cytometry was used to were profile for myeloid cell polarity/activation and results corroborated with indirect immunofluorescence assays. Simian human immunodeficiency virus (SHIV) DNA levels were measured with aid of the digital droplet polymerase chain reaction (PCR) assay. Luminex assays were then used to evaluate soluble plasma/CSF biomarker levels. Finally, changes in the fecal microbiome were evaluated using 16S rRNA on the Illumina NovaSeq platform.

**Results:**

Flow Cytometry-based semi-supervised analysis revealed that morphine exposure led to exacerbated M1 (CD14/CD16)/M2 (CD163/CD206) polarization in activated microglia that spanned across diverse brain regions. This was accompanied by elevated SHIV DNA within the sites of neurogenesis–HIP and SVZ. HIP/SVZ CD16+ activated microglia positively correlated with SHIV DNA levels in the brain (*r* = 0.548, *p* = 0.042). Simultaneously, morphine dependence depleted butyrate-producing bacteria, including *Ruminococcus* (*p* = 0.05), *Lachnospira* (*p* = 0.068) genera and *Roseburia_sp_831b* (*p* = 0.068). Finally, morphine also altered the regulation of CNS inflammation by reducing the levels of IL1 Receptor antagonist (IL1Ra).

**Conclusion:**

These findings are suggestive that morphine promotes CNS inflammation by altering receptor modulation, increasing myeloid brain activation, distorting gut-brain crosstalk, and causing selective enhancement of SHIV persistence in sites of neurogenesis.

## Introduction

Within the United States, the opioid epidemic results in over 125 daily deaths and approximately 70 billion dollars annually diverted toward criminal justice and healthcare systems (Florence et al., 2016; CDC/NCHS, 2018). Currently, the opioid crisis is embodied by recreational and overwhelming addiction to several drugs of abuse. Commonly used drugs of abuse range from prescription painkillers and naturally derived opioids such as morphine, codeine and opium (Rummans et al., 2018; Solimini et al., 2018). In addition, prescription synthetic opioids such as fentanyl, tramadol and carfentanil are also vastly misused (Pérez-Mañá et al., 2018). Interestingly, a significant proportion of opioid consumers are also infected with HIV-1. Morphine synergistically enhances viral loads and worsens HIV/SIV pathogenesis (Nath et al., 2000; Nath, 2002; Zou et al., 2011). In addition, morphine abuse results in remarkable neuropathogenesis characterized by neuronal dysfunction/degeneration that accelerates the occurrence of neuro-AIDS (Nath et al., 2000; Nath, 2002; Zou et al., 2011). Further, morphine administration also exacerbates alterations in gut homeostasis depicted by the disruption of the gastrointestinal epithelial barrier. Subsequently, this leads to elevated microbial translocation, microbial dysbiosis, and systemic immune activation (Meng et al., 2015a,b).

Morphine-mediated multiple organ dysregulation is fostered through several mechanisms, including the skewing of cytokines and chemokines. As a result, this facilitates increased expression of viral entry co-receptors, such as CCR5 and CXCR4 on target cells [CD4+ T cells, myeloid cells (monocytes, macrophages, and microglia)] (Guo et al., 2002; Steele et al., 2003; Kim et al., 2018). During inflammation, peripheral monocytes differentiate, acquiring proinflammatory phenotypes as they traffic and egress into tissues where they differentiate into macrophages (Shi and Pamer, 2011; Teh et al., 2019).

To gain access into the central nervous system (CNS), HIV-1 preferentially infects peripheral pro-inflammatory CD16+ monocyte subsets that subsequently breach the blood-brain barrier (Schechter et al., 2017). By compromising the blood-brain barrier, morphine increases the accessibility of virus-infected cells from the periphery into the CNS (Mahajan et al., 2008; Leibrand et al., 2019). Furthermore, SIV-infected rhesus macaques exposed to morphine exhibit exacerbated monocyte/macrophage influx into the CNS resulting in enhanced neuro-viremia (Bokhari et al., 2011). The compartmentalization of the CNS from the periphery limits the penetration of potent antiretroviral drugs. Consequentially, this offers a sanctuary for ongoing HIV/SIV replication, seeding of the viral reservoir, and persistent inflammation (Ellero et al., 2017). These events lead to a wide spectrum of cognitive impairments collectively termed as HIV-associated neurocognitive disorders (HAND) (Heaton et al., 2011; Farhadian et al., 2017).

Within the brain, HIV-1 principally infects the microglia (Rock et al., 2004) maintaining a steady state of quiescence and transcriptionally silent latency (Alvarez-Carbonell et al., 2019). However, HIV envelope gp120 and transactivator (Tat) proteins present in the CNS continue to promote neuronal damage despite ongoing viral latency (Tenneti and Lipton, 2000; Kaul and Lipton, 2005). Damaged neurons together with infiltrating proinflammatory macrophages release several proinflammatory cytokines, including interleukin (IL)-1β, IL-6, interferon-gamma (IFN-γ), and tumor necrosis factor-alpha (TNF-α) (Mizuno et al., 1994a,b, 2003). Resultantly, increased neurodegeneration, and augmented IL-1β- and TNF-α-dependent HIV reactivation within microglia occurs (Streit, 2006; Alvarez-Carbonell et al., 2017; Garcia-Mesa et al., 2017). Our laboratory has recently shown that following cART-mediated viral suppression, morphine-dependent SIV-infected rhesus macaques harbored elevated replication-competent reservoirs in the brain myeloid cells (Acharya et al., 2020).

The relative proportions of neuronal and myeloid cell lineages, varies within different brain regions (Ishino et al., 2017; Friedman et al., 2018; Tan et al., 2020). Given such variations, the effect of morphine on myeloid phenotypes and accompanying SIV reservoirs within diverse niches of the brain remains unknown. It is well recognized that the vagus nerve provides a conduit for bi-directional communication between the brain and the gut, highlighted by interjoining the enteric and CNSs (Breit et al., 2018). The production of neuro-immune mediators enables the brain to modulate intestinal functions (Carabotti et al., 2015; Breit et al., 2018). Reciprocally, the gut microbiota secretes metabolites such as short-chain fatty acids that foster optimal brain function (Bonini et al., 1997; Kimura et al., 2011; Dalile et al., 2019; Ma et al., 2019). Additionally, the specific composition of the microbiome can modulate crucial processes, such as neurogenesis in key brain compartments including the hippocampus (HIP) (Dinan and Cryan, 2017; Kelly et al., 2017). In fact, the disruption of the gut microbiome has been linked to several neurodegenerative disorders (Chen et al., 2021), such as Alzheimer’s disease (AD) (Kumar et al., 2016; Vogt et al., 2017), Parkinson’s disease (Laurent et al., 2013; Paiva et al., 2017), and multiple sclerosis (Jangi et al., 2016). Furthermore, morphine-mediated perturbation of the gut microbiome and simultaneous neuroinflammation have also been associated with dependence and tolerance (Lee et al., 2018). The complex interaction between the gut microbiome, inflammation, and brain-gut immune axis, however, remains poorly understood during HIV/SIV infection.

To address these knowledge gaps, we utilized a systems immunology approach using non-human primates (NHPs) as models for HIV infection. Compared to consenting human study participants, NHPs offer greater flexibility of interrogating changes in diverse tissue compartments (Winkler et al., 2012). HIV has a limited range of host infectivity and does not infect rhesus macaques (Iwanami et al., 2017). To bridge this limitation, we used an engineered chimeric simian human immunodeficiency virus (SHIV) AD8EO clone whose NF-κB binding sites within the long-term repeat (LTR) region were modified to enhance replication fitness (Dave et al., 2020). Following SHIV infection, we conducted comprehensive immune typing to understand phenotypic changes in myeloid cells found in various tissues. Simultaneously, we estimated viral DNA levels within diverse gut and brain regions and evaluated alterations in fecal microbiomes. In addition, changes in soluble factors in the cerebrospinal fluid (CSF) and plasma samples of SHIV-infected rhesus macaques were quantified.

Our findings suggested that morphine administration led to elevated myeloid cell activation across diverse brain regions and disrupted IL-1 regulation in the CNS by lowering the expression of CSF IL-1 receptor antagonist (IL-1ra). Further morphine increased viral persistence, particularly in sites of neurogenesis, while simultaneously disrupting the fecal microbiome. These observations have strong implications on future studies that will aim to dissect mechanisms by which morphine modulates the gut-brain axis.

## Materials and methods

### Rhesus macaques used and ethical approval for this study

Eight adult male (5–8 years of age) rhesus macaques accommodated within the Department of Comparative Medicine at the University of Nebraska Medical Center (UNMC) core animal facility were utilized for this study (Supplementary Table 1). Animals were pair-housed in steel cages within a temperature controlled (∼72° F) and light controlled (12-h light/dark cycle) room. Animals were routinely monitored by experienced staff twice a day. In addition to standard monkey chow (Purina, Gray Summit, MO, USA), their diet was supplemented with fresh fruits and vegetables. Environmental enrichment was also afforded by providing toys, foraging devices, and delicacies such as peanuts and cereals. At the terminal stage of this study, all animals were humanely euthanized by an overdose of a ketamine/xylazine mixture followed by transcardial perfusion with ice-cold PBS. All study procedures were approved by the Institutional Animal Care and Use Committee at UNMC. All study procedures were reviewed and approved by the UNMC IACUC protocol “15-113-01-FC.” This protocol was titled “The combinatorial effects of Opiates and promoter-variant strains of HIV-1 subtype C on neuropathogenesis and latency.”

### Morphine administration and subsequent simian human immunodeficiency virus infection

The overall study design is depicted in Supplementary Figure 1. This study included 8 rhesus macaques, four (*n* = 4) of which were intramuscularly injected with morphine (6 mg/kg/injection) twice a day on weekdays and one time on weekends (12 injections/week). Another four (*n* = 4) macaques received saline in parallel at same time points (12 injections/week) for 8 weeks. After 8 weeks, all rhesus macaques were intravenously inoculated with 200 TCID_50_ of SHIV AD8EO and its variants with varying 1–3 site of NF-KB sites in the promoter region (Dave et al., 2020). Thereafter, daily morphine or saline exposure was maintained until the end of the study (∼6–8 months). At necropsy, fecal samples, femoral blood, gut, liver, and whole brain tissue samples were collected.

### Preparation of peripheral blood mononuclear cells

Femoral blood was collected in K2-EDTA vacutainer tubes (BD, 367841). Within 4 h of collection, blood was centrifuged at 1,200 rpm for 20 min to separate the plasma. The remaining blood cells were layered over a Lymphoprep Density Gradient Medium from STEMCELL Technologies, Germany. Then, peripheral blood mononuclear cells (PBMCs) were isolated by density gradient centrifugal separation (Woollard et al., 2018).

### Preparation of liver mononuclear cells

Liver tissue was placed in RPMI immediately upon collection and finely chopped into 1 mm^2^ fine pieces using disposable scalpel blades. Following, the finely ground liver tissue (close to 10 g) was placed in digest media (20,000 U collagenase IV, 50 U DNase I, and 20 ml of DPBS) and incubated at 37°C for 30 min with occasional mixing in the Personal HybTm (Stratagene). The digested liver tissue was then filtered using 100 and 40 μm sterile cell strainers (Fisher Scientific, Pittsburgh, PA, USA).

### Preparation of gut mononuclear cells

For gut cell isolation, the digestion medium (10,000 U collagenase IV, 25 U DNAse I, and 10 ml DPBS) was prepared in advance for each tissue being processed. Tissue from the duodenum, ileum, and ascending colon were collected at necropsy. Approximately 10 g of lamina propria (LP) and muscularis (MUSC) mucosa sections were surgically excised for each gut section. Washes were then performed using DPBS in a petri dish, and tissues were carefully minced into 1 mm^3^ sections. Following this, 10 ml of digestion medium was added, and enzymatic digestion was performed for close to 1.5 h with frequent vortexing at 37°C. Then, 2 ml of FBS was added to each conical tube, and tissues triturated by gently pipetting up and down for additional homogenization. The digested gut tissue sections were then filtered using the 100 and 40 μm sterile cell strainers (Fisher Scientific, Pittsburgh, PA, USA). The filtrate was centrifuged at 1,200 rpm for 6 min and the resultant pellet resuspended in 5 ml of RPMI containing 20% FBS. The resuspended cells were then overlaid on a 60 and 30% Percoll gradient and centrifuged at 2,000 rpm for 30 min without braking. This was followed by targeting the cell layer found between the 30 and 60% interface comprising of lymphocytes and myeloid immune cells. The separated cells were washed with DPBS at 1200 rpm for 6 min and used for further analysis.

### Preparation of brain mononuclear cells

Following necropsy, portions of the whole brain tissue were dissected from the frontal lobe, cerebellum, medulla, putamen, HIP, and subventricular zone (SVZ). The examined tissue sections were washed with DPBS to remove any debris and minced into 1 mm^3^ pieces using scalpel blades and forceps. Then, 1–2 g of minced brain tissue was added to 50 mL conical tubes containing 10 mL of digestion medium (0.25% trypsin-EDTA + 25 U/mL DNase I in DPBS) and incubated for 1 h at 37°C with occasional mixing. After digestion, 2 mL of FBS was added to inhibit the digestive enzymes. The digestive tissue was triturated using a 25 mL pipette to enhance homogenization. Filtration was then performed using 100 and 40 μm sterile cell strainers (Fisher Scientific, Pittsburgh, PA, USA). The resultant filtrate was centrifuged at 1,600 rpm for 10 min at room temperature and the pellet was resuspended in DPBS. The resuspended cells were overlaid on a 60 and 30% Percoll gradient and centrifuged at 2,000 rpm for 30 min without braking. This was followed by targeting the cell layer found between the 30 and 60% interface comprising of lymphocytes and myeloid cells. The separated cells were washed with DPBS at 1,200 rpm for 6 min and were resuspended in Dulbecco’s Modified Eagle’s Medium (DMEM) media until further analysis.

### Flow cytometry of diverse tissue samples

#### Myeloid cell phenotyping in mononuclear cells obtained from diverse tissues

Tissue (blood, gut, liver, and gut) mononuclear cells were washed with DPBS and stained with zombie aqua live-dead stain to exclude dead cells. Then, FC receptor binding antibodies were added to minimize non-specific binding. After subsequent washes, a cocktail of surface receptor binding antibodies (listed in Supplementary Table 2A) suspended in BV buffer was added to the cells. Corresponding fluorescent minus one (FMO) tubes alongside compensation controls were also prepared simultaneously. Stained cells were fixed with 1% paraformaldehyde (PFA), and events were acquired using a Fortessa X450 instrument.

#### Automated and manual analyses of flow cytometry data

Compensated FCS3.0 files were exported from BD Facs Diva onto Flowjo Version 10. Manual gating was then performed using the gating strategies described in Supplementary Figures 2, 3. Automated analyses were performed using Flowjo Plugin Downsample version 3.0 to obtain similar events across several samples. After this, concatenations were carried out to obtain a single file. Later, the Flowjo Plugin Fast Fourier Transform-accelerated Interpolation-based t-SNE (FIt-SNE) was then executed on the resultant single concatenated FSC file at the following settings (t-SNE dimensions = 2, Nearest neighbors = Approximate, Perplexity = 20.0, and Maximum iterations = 3000) (Linderman et al., 2017).

#### Quantification of cell-associated simian human immunodeficiency virus DNA in various tissues

Digital droplet polymerase chain reaction (dd-PCR) was utilized to estimate the amount of SHIV DNA within the tissues. Genomic DNA was extracted using the AllPrep DNA/RNA Mini kit from Qiagen (Cat No./ID: 80204). The levels of genomic DNA were next evaluated using a GE SimpliNano drop spectrophotometer. Then, the Bio-Rad QX200 AutoDG digital droplet PCR system was used to carry out dd-PCR reactions. First, a reaction mixture comprising of: dd-PCR Supermix for probes (no dUTP), 10 μM forward primer, 10 μM reverse primer, 10 μM probe, DNA template, and RNase/DNase free water) was prepared. Then, 22 μl of the reaction mixture was added to each well of a 96-well plate and loaded onto a QX200 Droplet Generator for the generation of liquid droplets followed by heat sealing with a foil (Bio-Rad; Bio-Rad; Cat#181–4040). Amplification was carried out in a C1000 Touch thermal cycler (Bio-Rad, Hercules, CA, USA) under the conditions described in Supplementary Table 3. After this, the dd-PCR plates were placed in a QX200 droplet reader (Bio-Rad, Hercules, CA, USA) for droplet count and fluorescence measurements. With the aid of QuantaSoft software, the absolute quantity of DNA per sample (copies/μl) was determined after applying a fluorescence amplitude threshold to exclude negative droplets from the positive droplets containing the amplified products. The normalization for the conserved RPP30 gene was done to yield equivalent copies of SIV gag per million cells. Also, viral RNA levels were estimated using quantitative PCR (qPCR) assays as previously described (Acharya et al., 2020).

#### Indirect-immunofluorescence assay

Formalin fixed paraffin-embedded brain (HIP) tissues collected from six (11N074, 11N097, 12N060, 12N044, 12N015, and 13L126) different animals were grouped as SHIV+ saline (control *n* = 3) and SHIV+ morphine (infected *n* = 3). The selected tissues were then cut into 5 μm sections, deparaffinized in xylene and re-hydrated in descending grades of ethanol and deionized water. This was later followed by antigen retrieval in tris EDTA buffer (pH, 9.0), and blocking performed with ttPBS (1× PBS with 0.3% Tween 20 and 2 mM sodium azide) containing normal goat serum (S-1000, Vector laboratories, Burlingame, CA, USA). Next, the sections were incubated with unconjugated primary antibodies that comprised of: mouse anti-p27 SIV gag (clone 55-2F12, AIDS research and reference reagent program, Germantown, MD, USA), mouse anti-CD16 clone 2H7 (Novocastra, Newcastle, UK), rabbit CD163 Antibody (EDHu-1) (Novus biological, Centennial, CO, USA) and goat anti-Iba1 (ab5076) Abcam, Cambridge, Ma, USA at 4C overnight. Afterward, the tissues were washed and later stained with conjugated secondary antibodies such as: alexa488 goat anti-rabbit/goat anti-mouse, alexa546 goat anti-mouse and Dylight405 donkey anti-goat (1:200) for 1 h. The tissues were washed thrice with ttPBS, counter stained by DAPI 0.5 ug/ml for 10 min at room temperature, rinsed and mounted in prolong gold antifade reagent (Thermo Fisher, Waltham, MA, USA) (Dave et al., 2018). Images were acquired and quantified by using a Nuance fluorescence microscope equipped with Nuance software v 3.0.2 (Perking-Elmer Winter St Waltham, MA, USA). Quantification of positive cells was performed manually by counting positive staining per field in 10 random selected fields (Micci et al., 2014).

### 16S rRNA evaluation of the fecal microbiome

#### Genomic DNA extraction and 16S rRNA gene sequencing

Following the manufacturer’s instructions, DNA was extracted from thawed frozen (−80°C) fecal samples collected during necropsy using the Norgen Biotek Corp Stool DNA isolation kit (Catalog number: 27600). Using 341F/805R primers, a 465 bp amplicon was generated by targeting the V3 and V4 regions of the 16S rRNA gene. Then, sequencing was performed on the Illumina MiSeq platform (according to the manufacturer’s specifications) at LC Sciences to yield 250 bp paired-end reads in either direction (Johnson et al., 2021).

#### Microbiome data analysis

The obtained raw data files were demultiplexed, filtered, and processed to merge paired-end reads into a single continuous sequence tag. In contrast to exploiting sequence similarity within the Quantitative Insights Into Microbial Ecology (QIIME) platform, OTU Clustering was performed using the Divisive Amplicon Denoising Algorithm (DADA2). DADA2 reduces background noise and corrects sequencing errors by filtering, dereplication, chimeric filtering, and other methods. This improves data accuracy, species resolution, and reliability of results (Callahan et al., 2016). Taxonomy annotation was performed using the ribosomal database project (RDP) classifier tool for alignment with the corresponding OTU tags (Wang et al., 2007; Cole et al., 2014). Alpha diversity was then determined based on the number of OTUs/species (Chao-1 index) and uniformity (Shannon index). With the aid of both the R vegan package (Oksanen et al., 2016) and GraphPad Prism 9 software, principal component analysis (PCA) based on the weighted UniFrac distance matrix was used to evaluate Beta diversity (test for phylogenetic relatedness) (Motulsky, 2020). Bacterial taxa data were reformatted to serve as an input on the online Galaxy/Hutlab web platform for linear discriminant analysis effect size (LEfSe) analysis for taxa discrimination found in the two studied groups. For this analysis, the cut-offs used were linear discrimination analysis (LDA) ≥ 2 and *p*-values for Kruskal–Wallis and Pairwise Wilcoxon tests set to ≤0.05 (Hutlab; Segata et al., 2011).

#### Luminex high performance for multiple measures of soluble markers in plasma and cerebrospinal fluid

Plasma and CSF were collected at necropsy, frozen and shipped on dry ice for Luminex assays. Monoclonal antibodies were covalently bound to carboxylated Luminex beads following the carbodiimide procedure according to the manufacturer’s suggestion and washed and resuspended in PBS-0.5% Tween 20. Efficacy of coupling was confirmed with 1 μg/mL R-phycoerythrin goat anti-mouse IgG (H + L) antibody (Molecular Probes, Inc., Eugene, OR, USA). Commercial kits were run in individual plates with buffers and standards according to manufacturer’s directions. Following standardization, frozen samples were thawed quickly and 50 μL aliquots combined with coated beads. The acquisition gate was set between 8,000 and 13,500, and 100 events/region later acquired. Data were analyzed with the MasterPlex QT quantification software (MiraiBio Inc., Alameda, CA, USA). The following analytes were chosen for our analysis: macrophage inflammatory protein 1-alpha (MIP-1α), MIP-1β, Interleukin-6 (IL-6), interferon-γ-inducible protein 10 (IP-10), Interleukin-8 (IL-8), interferon-alpha (IFN-α), IFN-γ, Eotaxin, Interleukin-12p40 (IL-12p40), Interleukin-18 (IL-18), Interferon–inducible T-cell alpha chemoattractant (I-TAC), TNF-α, soluble CD40L (sCD40L), monokine induced by interferon-gamma (MIG), macrophage migration inhibitory factor (MIF), Interleukin-1 receptor a (IL-1Ra), Lymphatic Vessel Endothelial Receptor 1 (LYVE-1), Monocyte Chemoattractant Protein-1 (MCP-1), Regulated upon activation, normal T-cell expressed and presumably secreted (RANTES), Myeloperoxidase (MPO), Indoleamine 2, 3-dioxygenase (IDO), C-reactive protein (CRP) and perforin (Supplementary Table 2B). Measures for the levels of each analyte were interpolated from best fitting curves that were generated using commercial standards as previously described (Giavedoni, 2005).

### Statistical analysis

Prism V9.0 (GraphPad Software) and R version 3.4.3 were utilized for statistical analysis. Within-group comparisons (saline vs. morphine) were performed using the non-parametric Mann–Whitney *U*-test. Paired non-parametric tests were also performed using the Wilcoxon test. Also, grouped non-parametric tests were conducted using the Friedman test. For multiple comparisons, one-way analysis of variance (ANOVA) with Holm-Sidak *post hoc* testing was used. For multiple correlations, the cor function was used in R to generate a correlation matrix. *p*-values obtained within the correlation matrix were corrected for type 1 error using Holm’s correction. Following this, the R corrplot function was utilized to generate heatmaps. Also, the frequencies of flow cytometric measures from different brain regions were then plotted using the cerebroViz package (Bahl et al., 2017). Since the SVZ is not defined in cerebroViz, the best anatomical approximation, the caudate nucleus, was used to visualize this region.

## Results

### The dynamics of viral loads in the plasma and cerebrospinal fluid compartments of morphine-dependent vs. saline-exposed rhesus macaques

**FIGURE 1:**
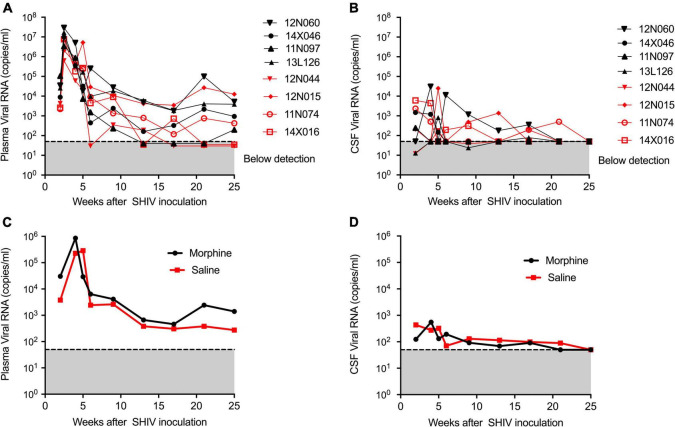
Kinetics of Plasma and CSF viral loads of morphine administered SHIVADE08 infected rhesus macaques. Plasma and CSF viral loads were quantified using a quantitative PCR (qPCR) assay. Longitudinal viral load measures in **(A)** plasma and **(B)** CSF of individual animals exposed to either morphine (red tagged) or saline (blue tagged). Geometric means of **(C)** plasma viral loads and **(D)** CSF viral loads were shown. The gray shaded zone displays the limit of detection of the assay (50 copies/ml). No statistical differences were observed between morphine vs. saline exposed SHIV infected rhesus macaques based on Mann–Whitney *U*-test comparisons at 0, 4, 8, 12, 16, 20, and 24-week timepoints.

During infection, the median peaks of plasma viral load in the morphine- and saline-treated groups were 1,338,500 and 6,430,000 copies per ml, respectively, (*p* = 0.220) (Figure 1A). Similarly, the median peaks of CSF viral load in the morphine and saline groups were 1,008 and 4,224 copies per ml, respectively, (*p* = 0.88) (Figure 1B). The longitudinal geometrical means of plasma and CSF viral loads of morphine and saline-exposed rhesus macaques are presented in Figures 1C,D with no statistical significance observed. Furthermore, we did not find significant differences in viral DNA and RNA levels in peripheral blood or several tissues examined (*p* > 0.05) (Supplementary Figure 2).

### Surface marker profile of the brain myeloid lineage in simian human immunodeficiency virus-infected saline or morphine-exposed rhesus macaques

**FIGURE 2:**
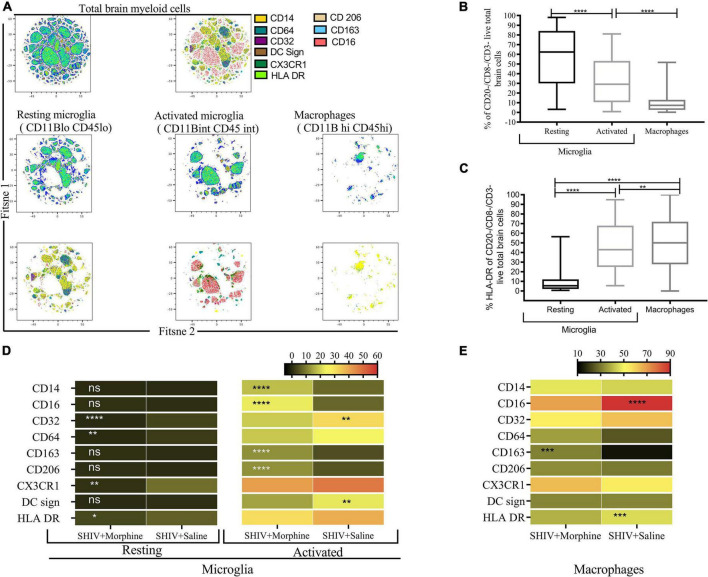
Flow Cytometric surface marker analysis reveals divergent brain myeloid phenotypes in SIV infected macaques exposed to either morphine or saline. **(A)** FitSNE projection of different macrophage/microglia phenotype markers expressed on total brain myeloid cells pooled from different brain regions in simian human immunodeficiency virus (SHIV) infected macaques (*n* = 8) exposed to either morphine or saline. **(B)** Percent frequencies of resting microglia, activated microglia and macrophages within total myeloid cells pooled from different brain regions as denoted by CD45 and CD11B discrimination. Data represent median ± range. **(C)** Evaluation of the extent of HLA-DR expression within resting microglia, activated microglia and macrophages pooled from diverse brain regions. Representative data denote median ± range. **(D)** Comparisons of % frequencies of CD14, CD16, CD32, CD64, CD163, CD206, CX3CR1, DC Sign, and HLA-DR in resting/activated microglia **(E)** macrophages in SHIV infected macaques treated with either morphine or saline. [*p < 0.05, **p < 0.01, ***p < 0.001, ****p < 0.0001 using the Wilcoxon test for paired non-parametric differences within groups **(B,C)**]. Mann–Whitney *U*-tests used for panels **(D,E)**.

The phenotype of brain myeloid cells is multi-dimensionally altered during different diseases, trauma, and neurodegeneration states (Herz et al., 2017). To gain insights into the global immune landscape of the predominant brain myeloid phenotypes, we profiled pooled cells isolated from different brain regions and evaluated their overall activation status, activation, frequency, and polarity. Thus an 18-parameter flow cytometry panel comprising size and complexity discrimination (FSC vs. SSC), dead cell exclusion (Zombie Aqua), dump lineage exclusion (CD3^+^, CD8^+^ NK^+^ and CD20^+^ cells), myeloid lineage markers (CD45 and CD11b), activation state (HLA-DR) and phenotypic markers (CD14, CD64, CD32, DC Sign, CX3CR1, CD206, CD163, and CD16) was utilized. Following event acquisition, manual gating was performed to obtain total brain myeloid cells after excluding dead cells and cells positive for the dump lineage markers (Supplementary Figure 3). Next, we used machine learning approaches to identify predominant myeloid cell phenotypes within brain cells. Based on the similitude of individual cell expression profiles, FitSNE projections of total brain myeloid cells segregated into distinct unbiased clusters of resting microglia, activated microglia, and macrophages based on CD45 and CD11b lineage discrimination (Figure 2A). As such, resting microglia (CD11b lo CD45 lo), activated microglia (CD11b int CD45 int), and macrophage (CD11b hi CD45 hi) subsets were noted. Our findings revealed that the resting microglia were the most predominant myeloid population in comparison to activated microglia and macrophages (*p* < 0.0001) (Figure 2B). Activated microglia expressed higher levels of HLA-DR than the resting microglia (Figure 2C). The resting microglia, as expected, demonstrated the lowest levels of all the phenotypic markers. Of note, the activated microglia of the morphine-exposed group expressed higher levels of CD14, CD16, CD163, and CD206 (*p* < 0.01 to *p* < 0.0001) compared to the saline-received group (Figure 2D). Similarly, macrophages of the morphine group expressed significantly higher levels of CD163 (*p* < 0.001). Unexpectedly, elevated levels of CD16 and HLA-DR were seen in the saline controls (*p* < 0.0001 and *p* < 0.001) (Figure 2E).

### Trends of elevated M1/M2 activated microglia span different brain regions of morphine-exposed rhesus macaques

**FIGURE 3:**
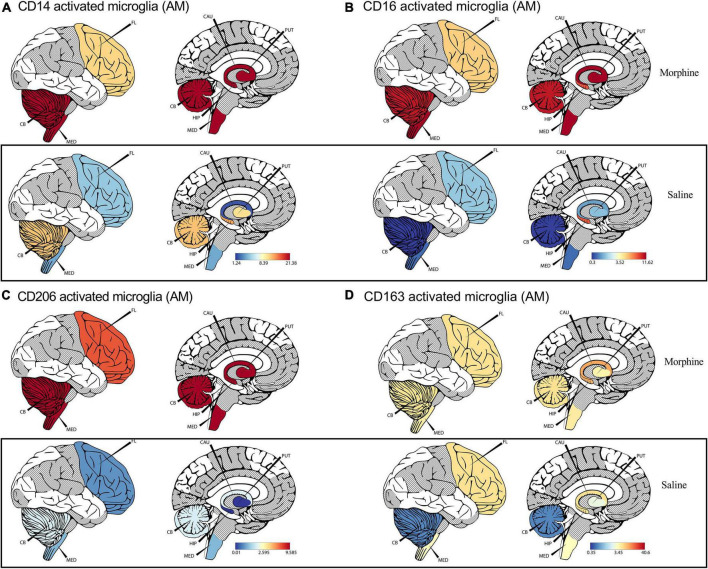
Morphine induces differential CD14, and CD163 profiles within diverse brain regions in simian human immunodeficiency virus (SHIV) infected rhesus macaques exposed to saline (*n* = 4) or morphine (*n* = 4). **(A)** CerebroViz visualization highlighting the anatomic distribution of **(A)** surface CD14, **(B)** surface CD16, **(C)** surface CD206, **(D)** surface CD163 on activated microglia contained within the frontal lobe (FL), cerebellum (CB), medulla (MED), Putamen (Put), Hippocampus (Hip), Caudate nucleus (CAU) in replacement of the subventricular zone (SVZ) activated microglia for visualization purposes.

Recent advances in cell sequencing, mass cytometry, and high parameter flow cytometry have furthered the understanding of the phenotypical heterogeneity of brain myeloid cells within the diverse regions of the brain (Mrdjen et al., 2018; Tan et al., 2020). To gain insights into the landscape of the differences in activated microglial cell activation across the different brain regions, we focused on profiling the expression of CD14, CD163, CD16, and CD206 markers. We observed a significant upregulation of CD14 on the surface of activated microglia, pooled from different brain regions of animals exposed to morphine (*p* < 0.0001) (Figure 2D). During evaluation of regional distribution, we noticed a trend of elevated surface CD14 expression on activated microglia was noticed within all studied regions (frontal lobe, cerebellum, medulla oblongata, SVZ, HIP and putamen) (Figure 3A). Similarly, these niches also contained increased levels of CD16 activated microglia and CD206 activated microglia in the morphine vs. saline exposed SHIV-infected rhesus macaques (Figures 3B,C) respectively. Remarkably, CD163 activated microglia levels were only visibly elevated in the cerebellum of morphine vs. saline exposed rhesus macaques (Figure 3D).

### Frequencies of CD16+ activated microglia correlate with gut/brain simian human immunodeficiency virus DNA levels

**FIGURE 4:**
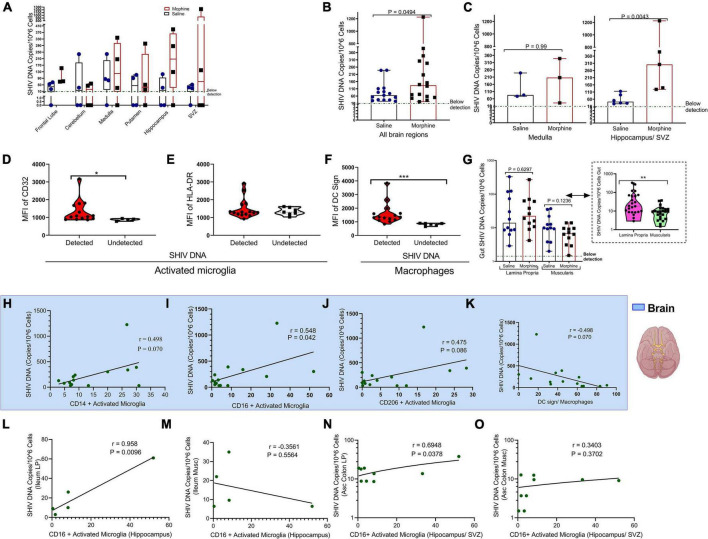
Relationship between CD16+ Activated Microglia and either gut or brain simian human immunodeficiency virus (SHIV) DNA. **(A)** Levels of SHIV DNA within the frontal lobe, cerebellum, medulla, putamen, hippocampus, and subventricular zone (SVZ) in morphine vs. saline exposed rhesus macaques. **(B)** Comparison of levels of SHIV DNA pooled within all the brain regions after adjusting for regions in which the virus was not detected. **(C)** Levels of SHIV DNA within the brain stem (medulla) and sites of neurogenesis (hippocampus/SVZ) in morphine vs. saline-treated rhesus macaques. **(D)** Mean Fluorescence Intensity (MFI) of CD32 **(E)** HLA-DR in activated microglia and **(F)** DC Sign in macrophages found within niches of detected and undetected SHIV DNA across the total brain. **(G)** Levels of SHIV DNA within the lamina propria and muscularis mucosa with further stratification of saline and morphine groups. All data represent median ± range in Panels **(A–G)**. **(H)** Association of brain SHIV DNA with CD14+ activated microglia **(I)** CD16+ activated microglia **(J)** CD206 activated microglia, and **(K)** DC Sign macrophages pooled form all brain regions in which SHIV DNA was detected. **(L)** Association between hippocampus CD16+ activated microglia and either SHIV DNA in ileum lamina propria (LP) or **(M)** ileum muscularis (Musc). **(N)** Relationship between CD16+ activated microglia found in neurogenic sites (hippocampus/SVZ) in ascending colon (Asc Colon) LP and **(O)** Asc Colon Musc. (*p < 0.05, **p < 0.01, ***p < 0.001). Mann–Whitney *U*-tests were used for significance in panels **(B–F)**. For panel **(G)**, multiple comparisons were evaluated using one-way ANOVA with Holm-Sidak *post hoc* testing.

Within the diverse regions of brain examined, SHIV DNA levels were highly variable across different sites evaluated. However, in some regions no viral DNA was detected (Figure 4A). Upon normalizing and adjusting for sites where no DNA was detected, morphine exposure was associated with increased levels of SHIV DNA (Figure 4B). A region-based perspective of the modulation of SHIV DNA by morphine revealed no changes in the levels of viral DNA within the brain stem (medulla). Importantly, the bulk of morphine-induced enhancement in the levels of viral DNA appeared in sites of neurogenesis (HIP and the SVZ), (*p* = 0.0043) (Figure 4C). Myeloid cells have been reported to be the principal HIV/SIV DNA reservoirs in the brain (Wallet et al., 2019). Therefore, we next compared the profiles of activated microglia and macrophages at the sites where the SHIV DNA was detected vs. undetected. The levels of CD32 on activated microglia were elevated in the brain regions where SHIV DNA was detected (Figure 4D). No differences in HLA-DR expression were noted (Figure 4E). Similarly, the levels of DC sign on macrophages were also enhanced in SHIV DNA positive vs. negative brain regions (Figure 4F). In the gut, higher levels of the SHIV DNA were found in the LP vs. the muscularis (*p* = 0.01) (Figure 4G). Remarkably, in the brain, multiple markers of microglial polarity were associated with SHIV DNA. Spearman rank correlation analysis showed that increases in CD16+ activated microglia were accompanied by a significant elevation of brain SHIV DNA (*r* = 0.548, *p* = 0.042) (Figure 4I). Other markers of myeloid polarity expressed on activated microglia, such as CD14 (*r* = 0.498, *p* = 0.070), CD206 (*r* = 0.475, *p* = 0.080), and DC Sign+ brain macrophages (*r* = 0.498, *p* = 0.070) had borderline associations with the brain SHIV DNA (Figures 4H,J,K). Lastly, the viral DNA levels within the ileum LP (*r* = 0.958, *p* = 0.0096) (Figure 4L), not ileum muscularis (*r* = −0.3561, *p* = 0.5564), (Figure 4M) were positively correlated with the CD16+ activated microglia present in the HIP (*r* = 0.958, *p* = 0.0096), (Figure 4L) as opposed to ileum muscularis SHIV DNA (*r* = −0.3561, *p* = 0.5564), (Figure 4M). Finally, % CD16 activated microglia found in the neurogenic niches (HIP and SVZ), were associated with increasing SHIV DNA within the LP (*r* = 0.6948, *p* = 0.0378), (Figure 4N) unlike in the muscularis (*r* = 0.3403, *p* = 0.3702), (Figure 4O) of the ascending colon.

### Immunofluorescence corroborates findings indicating that morphine exposure leads to elevated expression of diverse myeloid markers that co-stain with SIV p27

To corroborate our earlier findings using another method (immunofluorescence) to complement our flow cytometry findings, we observed that within the HIP tissue, morphine exposure led to increased expression of CD163 (*p* = 0.039). In addition, the augmented expression of CD163 was also associated with elevated co-staining with SIVp27 (Supplementary Figures 4A,E). Similarly, morphine exposure also led to increased expression of CD206 (*p* = 0.0015) (Supplementary Figures 4B,E). Rhesus macaques exposed to morphine also had a marginally statistically significant increased expression of the myeloid activation marker Iba-1 (*p* = 0.066) that was also shown to increasingly colocalize with SIVp27 (Supplementary Figures 4C,E). Lastly, the expression of CD16 was not statistically different in morphine vs. saline exposed SHIV infected rhesus macaques despite the intensified staining observed in morphine treated animals (Supplementary Figure 4D).

### Exposure to morphine was associated with community level depletions of the microbiome in simian human immunodeficiency virus infected rhesus macaques

**FIGURE 5:**
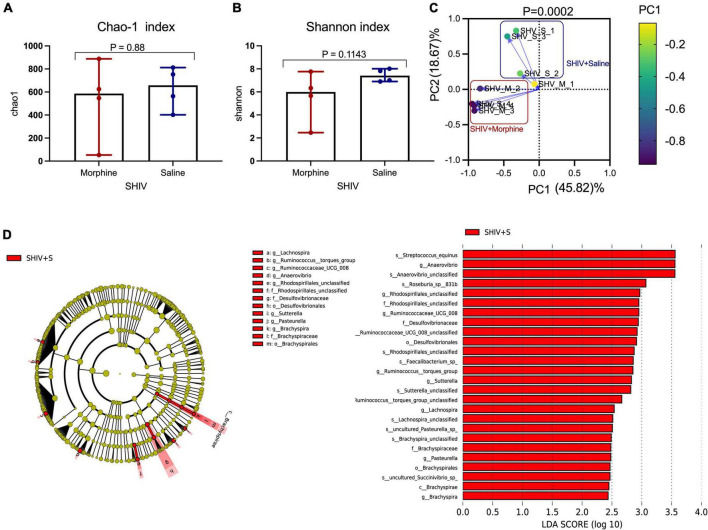
Exposure to morphine disorientates gut microbiome homeostasis in simian human immunodeficiency virus (SHIV) infected rhesus macaques. Differences in **(A)** Chao-1 index **(B)** Shannon index as measures pf Alpha diversity in rhesus macaques exposed to either morphine or saline. Mann–Whitney *U*-tests were used to evaluate differences within the two groups in panels **(A,B)**. Data represent median ± range in panels **(A,B)**. **(C)** Weighted UniFrac Principal component analysis showing distinct clustering of SHIV + morphine animals vs. SHIV + saline animals. Variance was reported following the use of PCA in panel **(C)**. **(D)** Cladogram indicating significantly different taxa calculated using linear discriminant analysis effect size (LEfSe) analysis for microbiome discrimination at the community level (class, family, genus, and species) of SHIV + saline (red) vs. SHIV + morphine (blue) rhesus macaques. Side by side, corresponding tabulated linear discriminant analysis (LDA) are also presented with the logarithmic threshold score being set at 2.0. Using the Kruskal–Wallis and pairwise Wilcoxon tests, *p* < 0.05 set as cut-off for differences within the studied groups.

Key genera of the gut microbiome, collectively termed as the psychobiome (Bates, 2021), are postulated to produce secondary metabolites that foster optimal brain activity and modulate intestinal myeloid lineage activation (Carabotti et al., 2015; Ji et al., 2016). Upon analyzing for average species diversity within the gut habitat (alpha diversity), we found that there were no differences in the number of OTUs/species (Chao-1 index), (*p* = 0.88) (Figure 5A) and uniformity of species (Shannon index), (*p* = 0.1143) within morphine-exposed and saline-received rhesus macaques (Figure 5B). Upon evaluating for changes within microbial populations, beta diversity using the principle component analysis of unweighted UniFrac distance matrix revealed that the OTUs of morphine exposed rhesus macaques clustered distinctly from the saline recipient animals (*p* = 0.0002 based on multiple linear regression) (Figure 5C). Using LEfSE, we observed a unidirectional change that saline exposed animals contained elevated OTUs of families *Brachyspiraceae* and *Desulfovibrionaceae*, genera *Ruminococcus torques* group, *Ruminococcaceae* UCG_008, *Anaerovibrio* and *Lachnospira* [LDA score (log 10) > 2] in comparison to morphine exposed rhesus macaques (Figure 5D).

### Morphine selectively depletes key genera and species of the fecal microbiome

**FIGURE 6:**
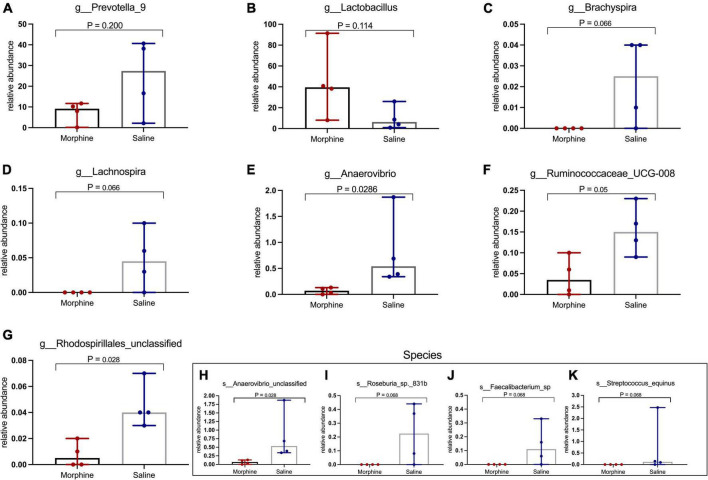
In simian human immunodeficiency virus (SHIV) infected rhesus macaques, exposure to morphine leads to reduced distinct genera and species of the fecal microbiota. Genus and Species differences within the fecal microbiomes as detailed by **(A)** *Prevotella-9* genus, **(B)** *Lactobacillus* genus, **(C)** *Brachyspira* genus, **(D)** *Lachnospira* genus, **(E)** *Anaerovibrio* genus, **(F)** *Ruminococcaceae* UCG-008, **(G)** *Rhodospirillales* unclassified, **(H)** *Anaerovibrio* species, **(I)** *Roseburia_sp_831b*, **(J)** *Faecalibacterium* species, **(K)** *Streptococcus equinus* species of SHIV + morphine animals vs. SHIV + saline rhesus macaques. [*p* < 0.05 deemed significant while borderline significance (*p* = 0.05 to *p* = 0.07), all obtained using the Mann–Whitney *U*-test for non-parametric differences within the two classified groups].

Morphine did not induce changes in key HIV-associated genera, such as *Prevotella-9*, (*p* = 0.200) (Figure 6A), most homologous to *Prevotella copri*, the pathobiont in human microbiomes associated with inflammatory diseases, and *Lactobacillus* (*p* = 0.114) (Figure 6B), majorly considered anti-inflammatory (Dillon et al., 2016). Further, morphine administration resulted in a reduction of *Brachyspira* (Figure 6C) and *Lachnospira* (Figure 6D), although these differences were not significant (both *p* = 0.0666). The relative abundance of *Anaerovibrio* (*p* = 0.028) (Figure 6E) and *Ruminococcaceae* UCG_008 (*p* = 0.05) (Figure 6F) were significantly lower in the morphine-treated vs. saline recipients. Furthermore, *Rhodospirillales* unclassified species were also significantly reduced in the morphine-treated vs. saline administered rhesus macaques (*p* = 0.028) (Figure 6G). Lastly *Anaerovibrio* unclassified species (*p* = 0.028) (Figure 6H) and *Roseburia* species (*p* = 0.008) (Figure 6I) were significantly depleted. There were no significant depletions in *Faecalibacterium* species (Figure 6J) and *Streptococcus equinus* species (Figure 6K) in the morphine-exposed vs. the saline group (both *p* = 0.068).

### Morphine disrupts the levels of key cytokines and chemokine receptor levels in the cerebrospinal fluid and plasma of rhesus macaques

**FIGURE 7:**
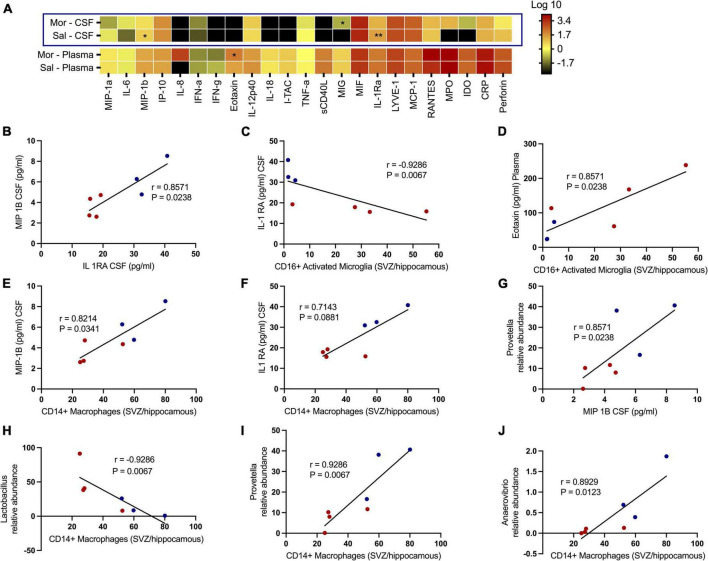
Morphine dysregulates chemokine and inflammatory cytokine receptors in plasma/the CNS that intercorrelate with CD16+ activated microglia/CD14+ macrophages in sites of neurogenesis and specific genera of the fecal microbiome further offering credence to morphine’s possible effects on interactions of the gut-brain axis. **(A)** Changes within the CSF and plasma soluble factor profiles of simian human immunodeficiency virus (SHIV) infected rhesus macaques exposed to either morphine or saline. Multiple correlations between diverse parameters of the fecal microbiome, brain immune cells and CSF/plasma soluble factor indices as shown by relationships between **(B)** MIP-1B CSF (pg/ml) and IL-1 RA CSF (pg/ml). **(C)** IL-1 RA CSF (pg/ml) and CD16+ activated microglia [subventricular zone (SVZ)/hippocampus]. **(D)** Eotaxin plasma (pg/ml) and CD16+ activated microglia [(SVZ)/hippocampus]. **(E)** MIP-1B CSF (pg/ml) and CD14+ macrophages [(SVZ)/hippocampus]. **(F)** IL-1 RA CSF (pg/ml) and CD14+ macrophages [(SVZ)/hippocampus]. **(G)** *Prevotella* relative abundance and MIP-1B CSF (pg/ml). **(H)** *Lactobacillus* relative abundance and CD14+ macrophages [(SVZ)/hippocampus]. **(I)** *Prevotella* relative abundance and CD14+ macrophages [(SVZ)/hippocampus]. **(J)** *Anaerovibrio* relative abundance and CD14+ macrophages [(SVZ)/hippocampus]. *p* < 0.05 is considered statistically significant. (Red dot points signify morphine treated SIV infected rhesus macaques whilst blue dot points are saline treated SIV infected rhesus macaques). (*p < 0.05, **p < 0.01, using the Spearman’s rank correlation test).

We additionally tested how exposure to morphine affected soluble factors associated with chemotaxis and inflammation underlying SHIV infection (Figure 7A). No significant differences were observed with several proinflammatory cytokines, such as CRP, TNF-α, IL-6, IL12 p-40, IFN-α, and IFN-γ. Remarkedly, the levels of MIP-1β (*p* = 0.0469) and IL-1Ra (*p* = 0.0014) in the CSF were elevated in saline- vs. morphine-treated rhesus macaques. Conversely, the MIG levels were elevated in the CSF of SHIV-infected rhesus macaques that received morphine. In the plasma of SHIV-infected rhesus macaques, the Eotaxin (*p* = 0.0387) levels were similarly elevated in the morphine- vs. saline-treated animals (Figure 7B). Having observed a reduction in the levels of MIP-1β and IL-1Ra in morphine-exposed animals, we asked if an association existed between these analytes and found a strong positive correlation (*r* = 0.8571 and *p* = 0.0238). We extended the analysis of associations between key cell types localized within brain regions where high levels of SHIV persistence were observed. CD16+ activated microglia (SVZ/HIP) were strongly associated with IL-Ra in the CSF (*r* = −0.9286 and *p* = 0.0067) (Figure 7C) and Eotaxin in the plasma (*r* = 0.8571 and *p* = 0.0238) (Figure 7D). CD14+ macrophages (SVZ/HIP) were strongly associated also with MIP-1β (*r* = 0.8214 and *p* = 0.0341) (Figure 7E), and IL-1Ra (*r* = 0.7143 and *p* = 0.0881) in the CSF (Figure 7F). *Prevotella* relative abundance in the fecal microbiome was also positively correlated with MIP-1β (*r* = 0.8571 and *p* = 0.0238) (Figure 7G). Evaluation of associations between the key genera of the fecal microbiome and the CD14+ macrophages (SVZ/HIP) highlighted the presence of strong correlations between this cell subset and *Lactobacillus* relative abundance (*r* = −0.9286 and *p* = 0.0067) (Figure 7H), *Prevotella* relative abundance (*r* = 0.9286 and *p* = 0.0067) (Figure 7I), and *Anaerovibrio* (*r* = 0.8929 and *p* = 0.0123) (Figure 7J), respectively.

## Discussion

Our results present novel findings on how morphine affects brain myeloid lineage polarization and SHIV persistence in different brain regions. First, we reveal that morphine increases microglia activation traversing M1/M2 polarity within diverse brain regions, while enhancing SHIV persistence particularly in sites of neurogenesis. In tandem, morphine also depletes crucial genera of the gut microbiome, which could affect optimal brain function. Finally, we provide extra cues as to how morphine further disorganizes gut-brain crosstalk during SHIV infection.

Similar to several previous reports, we found that resting microglia were the predominant myeloid phenotype in the brain and exhibited a quiescent state. This was characterized by the low expression of cell surface markers, including the lineage markers CD45, CD11B, and the myeloid pan activation marker HLA-DR. Notably, activated microglia/macrophages exhibited elevated levels of these surface molecules (Nimmerjahn et al., 2005; Conrad and Dittel, 2011; Ponomarev et al., 2011; Jurga et al., 2020). Worse still, the increased surface expression of mu-opioid receptors on activated microglia makes this cell type more susceptible to alterations following morphine exposure (Maduna et al., 2019). Hence, we noticed increased plasticity of activated microglia, as shown by elevated surface pro-inflammatory M1 markers (CD14, CD16) and anti-inflammatory M2 markers (CD163 and CD206) in SHIV infected rhesus macaques exposed to morphine. Similarly, morphine was shown to increase myeloid cell activation traversing M1/M2 phenotypes in tissues of cancer patients (Tu et al., 2020).

Interestingly, CD14+ expression on microglia exacerbates the brain’s inflammatory milieu, as demonstrated by increased plaques in AD (Liu et al., 2005; Jurga et al., 2020). As predicted, the elevation of CD16 (FcγRIIIA) that promotes pro-inflammatory signaling was accompanied by a concomitant reduction of CD32 (FcγRII) that facilitates anti-inflammatory signaling.

Accumulation of CD163 and CD206 alternative microglia could serve as a means to resolve the burgeoning morphine-mediated inflammatory damage involving phagocytosis of debris, and clearance of haptoglobin complexes (Biswas and Mantovani, 2010; Etzerodt and Moestrup, 2013; Park et al., 2016; Ohgidani et al., 2017). Within dissected brain regions, the observed heterogeneity of morphine-mediated myeloid activation mirrors previous publications that report regional differences in myeloid cell morphology and spatial diversity (Bachiller et al., 2018; Tan et al., 2020). Increased CD14+ and CD163+ expression within the frontal lobe and the cerebellum of morphine-dependent animals could hint at more intense inflammation-induced atrophy within these regions (Hoyer et al., 2017; Israel et al., 2019). Collectively, this further underscore morphine’s myeloid skewing toward increased neuro-inflammation as further confirmed by our imaging studies that showed elevated myeloid activation based on Iba-1and distorted M1/M2 phenotypes based on CD16, CD163, and CD206 (Sudduth et al., 2013; Hovens et al., 2014; Walker and Lue, 2015).

The decreased levels of the chemokine MIP-1β observed within the CSF of morphine-dependent rhesus macaques is consistent with previous findings (Mahajan et al., 2002). These studies reported that morphine lowers expression of MIP-1β while chemokine receptor CCR5 expression in microglia, hence facilitating viral entry (Mahajan et al., 2002). Likewise, lower levels of CSF IL-1Ra could have implications of less readily available receptor levels, which in turn, could block the binding of the highly inflammatory cytokine IL-1. Subsequently, this may result in the facilitation of morphine-mediated neuroinflammation, further fueling the establishment of neuro-SHIV propagation and persistence (Murray et al., 2015).

The finding that morphine exposure enhances the levels of SHIV DNA at the sites of neurogenesis (HIP and SVZ) and the brain stem (medulla) alludes to the seeding of the viral reservoir in selective brain regions. Along these lines, Perez et al. (2018) and Putatunda et al. (2019) found that viral DNA primarily localizes to similar brain regions (Perez et al., 2018; Putatunda et al., 2019). Myeloid cells have been proposed as the principal viral reservoirs in tissues (Rodrigues et al., 2017; Ko et al., 2019). Remarkably, in sites where we detected viral DNA, increased expression of DC sign+ on brain macrophages could hint at increased viral permissivity (Lee et al., 2001). The significant association between CD16+ activated microglia and brain SHIV DNA further highlights an association between immune activation and viral persistence in the brain. Our imaging findings are also consistent with the previous reports which showed that CD163, CD206 and CD16 microglia/macrophages harbored HIV DNA (Fischer-Smith et al., 2008; Nowlin et al., 2015; Ko et al., 2019). As such, this further builds on evidence that brain myeloid cells serve as viral reservoirs (Abreu et al., 2019; Mitchell et al., 2019).

The observation that morphine increases viral persistence in sites of neurogenesis points to healthy neurons being crucial for maintaining latency in microglia. Recent reports suggest that optimal communication between healthy neurons and microglia through the mediation of receptors such as CD200 and CX3CL1 are required to maintain pro-viral DNA transcriptionally silent (Alvarez-Carbonell et al., 2020). Further, morphine also alters the homeostasis of the CNS through elevated inflammation and chronic myeloid activation. In combination, this could enhance the generation of damaged neurons which could concomitantly lead to increased amounts quantities of SHIV DNA in sites of neurogenesis.

The observation that increased levels of CD16+ activated microglia at the neurogenic niches were associated with the presence of viral DNA in the ileum LP and the ascending colon LP hints at possible gut-brain crosstalk. Based on the existence of a gut-brain axis, metabolites such as SCFAs secreted by the gut microbiome may influence CNS inflammation and shape microglial maturation (Erny et al., 2015; Meneses et al., 2016; Mertsalmi et al., 2017; Abdel-Haq et al., 2019). It is possible that SIV-associated gut dysbiosis compounded by the effects of morphine could crucially contribute toward CNS dysregulation, particularly at the sites of neurogenesis. The influence of the fecal microbiome on brain functions, such as neurogenesis, was ascertained after fecal transplants obtained from old mice promoted hippocampal neurogenesis in germ-free mice (Kundu et al., 2019). Our findings morphine perturbs the diversity of the gut microbiota at the community level agrees with previous reports that show that morphine distorts the gut bacterial microbiome (Zhang et al., 2019; Johnson et al., 2021). Further, our findings agree with the previous reports that show that morphine exposure causes the depletion of SCFA-producing bacteria (Sindberg et al., 2019; Gicquelais et al., 2020; Johnson et al., 2021).

We noted that specific butyrate secreting bacterial species, such as *Roseburia_sp_831b* species together with *Ruminococcus* and *Lachnospira* majorly represented the depleted SCFA producing bacteria. The secreted metabolites released by these genera are crucial for the reduction of the gut inflammation (Parada Venegas et al., 2019), production of antimicrobial peptides (AMPs) (Hillman et al., 2020), and the maintenance of the gut barrier function (Peng et al., 2009; Kelly et al., 2015). Unlike our previous study, where we observed that *Prevotella-9* replaced the butyrate-producing bacteria in morphine-exposed, ART-treated, and SIV infected rhesus macaques, we did not notice any fluctuations in *Prevotellaceae* and an associated α-diversity (Johnson et al., 2021). In this study we observed a strong positive association between pro-inflammatory *Prevotella-9* and the CSF MIP-1β/CD14+ macrophages (SVZ/HIP) as opposed to a strong negative correlation between anti-inflammatory *Lactobacillus* and CD14+ macrophages (SVZ/HIP), alluding to elevated gut inflammation and dysbiosis affecting CNS inflammation. These findings similarly highlight processes involved in the loss of gut barrier function, leading to concomitant microbial translocation and the systemic immune activation/inflammation previously reported during morphine enhanced HIV pathogenesis (Meng et al., 2013; Banerjee et al., 2016). Strong associations between proinflammatory gut microbiota and CD14+ macrophages in the SVZ suggests that the expansion of this population is reliant on a highly proinflammatory gut milieu. Collectively, these interrelationships offer additional credence to morphine’s ability to perturb the previously reported multiple systems cross-talk that occurs between the gut microbiota, enteric-nervous and brain systems (Lee et al., 2018; Cryan et al., 2019; Zhang et al., 2019).

In summary, we posit that morphine-associated CNS dysregulation is sustained by the loss of the feedback control of pro-inflammatory cytokines such as IL-1, the depletion of the psychobiome and the dysregulation of the gut-brain axis crosstalk that exacerbates the reactivation of the proviral reservoir. Consequentially, this results in a vicious cycle where the recently released viruses infect new and susceptible microglial cells, causing fluctuations in the size of the viral reservoir. Future studies on limiting neuro persistence in morphine abusers should aim at limiting morphine-enhanced defective neuron-microglia crosstalk by targeting receptors such as the CD200 receptor and CX3CL1. In addition, metabolomic screens should be carried out to understand how morphine affects key metabolites involved in optimal crosstalk between the gut microbiome and HIP (Liu et al., 2021). Lastly studies that look at the contribution of other sections of the microbiome such as the virome and fungi toward gut-brain axis crosstalk should also be considered (Townsend et al., 2021; Du Toit, 2022; Leonardi et al., 2022).

**FIGURE 8:**
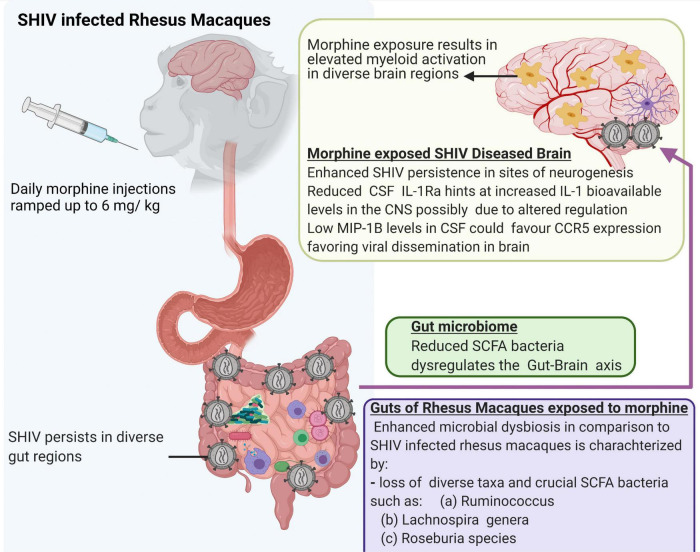
Summary of multiple effects of morphine on the gut-brain axis of simian human immunodeficiency virus (SHIV) infected rhesus macaques.

Lastly, we acknowledge that a small number of animals were utilized in the present study. In addition, our ability to evaluate the viral reservoirs, especially in the gut compartment is limited by the fact that the study animals were not exposed to cART. Despite these limitations, our study offers new leads suggesting that morphine increases the seeding of the neuro-SHIV reservoirs primarily at the sites of neurogenesis. This is simultaneously accompanied by the elevated brain myeloid cell activation with the M1/M2 polarization spanning across diverse brain regions and a skewed regulation of neuroinflammation. Furthermore, our findings provide novel insights into how morphine affects the gut-brain axis and offer a blueprint that could guide future mechanistic studies focused on dissecting key processes by which HIV affects multiple organ cross-talk during substance abuse (Figure 8).

## Data availability statement

Microbiome data presented in this study are deposited in the NCBI BioProject database, accession number PRJNA870584. Source code for generating Figure 3 can be found on: https://github.com/aomalla123/Cerbroviz-code/blob/main/README.md.

## Ethics statement

The animal study was reviewed and approved by Institutional Animal Care and Use Committee University of Nebraska Medical Center.

## Author contributions

OO, SJ, MB, and SNB designed the study. OO, MB, and SNB designed the flow cytometry panel. OO, SJ, and MB carried out sample acquisition and performed analysis of related data. KP, MT, SJ, and AA carried out estimation of the SIV viral reservoir within diverse gut and brain regions. OO, SJ, and SU were involved in tissue processing. VT performed the immunofluorescence analysis. SJ performed the DNA extraction from fecal material. SJ and OO were involved in microbiome data analysis. LG, MB, and SJ were involved in Luminex evaluations. SC performed macaque studies and morphine administration. UR, SJB, and SNB designed the parent study and solicited funds to support this work. All authors are read, edited, and approved the manuscript.

## Supplementary material

Supplementary Figure 1 Study design involving treatment of rhesus macaques and intricate detailing of experiments utilized for this study. During the entire course of the study, eight rhesus macaques were given either morphine (*n* = 4) at 6 mg/kg and saline (*n* = 4) twice daily and once on weekends. After 8 weeks, rhesus macaques were infected with SHIVADE08. Using fecal samples, the 16s rDNA fecal microbiome was studied using the illumine sequencing platform. Similarly, flow cytometry was utilized to interrogate changes in myeloid modifications found in the brain, gut, and liver. Levels of SHIV DNA were estimated using the digital droplet polymerase chain reaction (dd-PCR) assay across different regions of gut and brain tissues.

Supplementary Figure 2 Viral dynamics in peripheral blood and diverse tissues. Differences in Gag DNA levels found in **(A)** PBMC CD4+ T-cells **(B)** Lungs **(C)** Spleen in eight SHIV infected rhesus macaques exposed to either morphine or saline. Differences in Gag RNA levels found in **(E)** PBMC CD4+ T-cells **(F)** Lungs **(G)** Spleen in eight SHIV infected rhesus macaques exposed to either morphine or saline.

Supplementary Figure 3 Brain gating strategy used to interrogate brain myeloid (microglia and macrophages) found in diverse brain regions. Various brain regions were dissected and tissue digestion carried out using DNAse and Trypsin and obtained single-cell suspensions analyzed using the BD Fortesssax450. Detailed gating strategy involving an FSCA vs. Time to check the stability of the flow and obtain events only when an expected stream flows were obtained. Using Pulse Geometry scales comprised of FSC-H vs. FSC-A and SSC-H vs. SSC-A dot plots, we excluded doublets. Next, dump gates were used to eliminate unwanted populations. CD8α+ cells and Live Dead exclusion plus CD3+ T-cell removal guided the exclusion of CD3+ T-cells, NK cells, and dead cells. Next, the CD20 dot plots guided the removal of B-cells. Collectively, this ensured that the major populations studied in Brain cells were majorly myeloid cells. CD45 vs. CD11B plots were utilized to guide segregation of resting/activated microglia in addition to macrophages. Fluorescence minus one (FMO) controls were used to guide the proper placement of gates of diverse myeloid cell markers within the resting microglia, activated microglia and macrophages.

Supplementary Figure 4 Surface expression of diverse myeloid markers in the hippocampus of SHIV-infected saline or morphine-exposed rhesus macaques. Immunofluorescence images of the hippocampus was stained with different macrophage markers: **(A)** CD163 and **(B)** CD206 (green) together with SIV-p27 (red) and DAPI for nucleus was visualized by fluorescence microscope. **(C)** Single and combined staining for Iba 1 (blue), SIV-p27 (red) and merged co-expression of Iba 1and SIV-p27. **(D)** Single stained CD16, SIV p27 and CD16 (green) co-expressed with p27 in the brain of one representative rhesus macaque. The upper panel indicates SHIV + saline while panel **(E)** represents SHIV + morphine respectively. Quantitative analyses were done by two groups each *n* = 3 showing the number of cells per mm^2^ of tissue that stained positively for the markers of interest (*p* < 0.05 signifies statistical significance as carried out using unpaired *T*-tests).
