# Supplementary figures and images for: Systems biology analyses reveal enhanced chronic morphine distortion of gut-brain interrelationships in simian human immunodeficiency virus infected rhesus macaques
Source: Front Neurosci. 2022 Oct 13;16:1001544. doi: 10.3389/fnins.2022.1001544 (PMC9613112; doi:10.3389/fnins.2022.1001544)

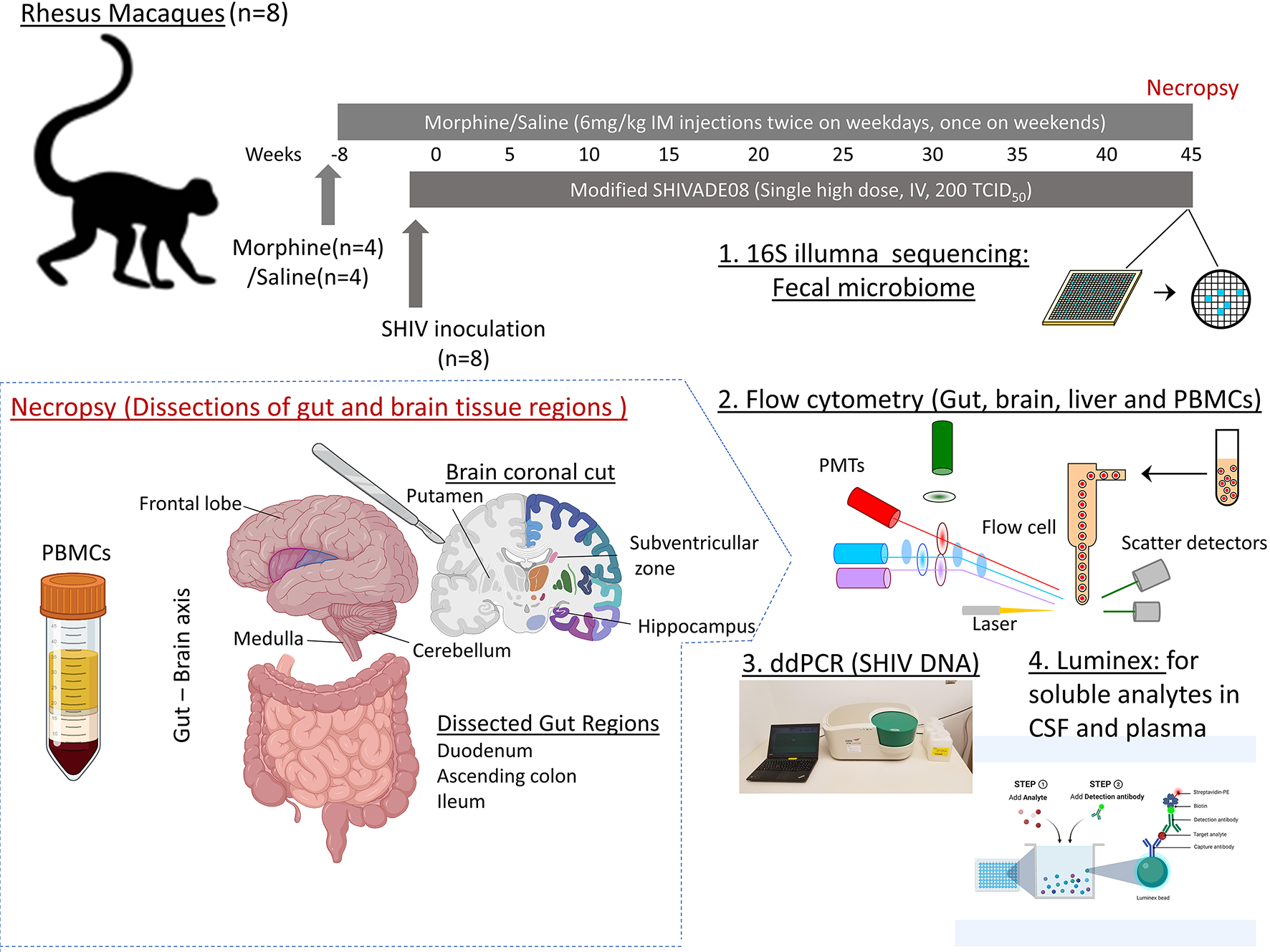

Supplement: Supplementary Figure 1 — Study design involving treatment of rhesus macaques and intricate detailing of experiments utilized for this study. During the entire course of the study, eight rhesus macaques were given either morphine (n = 4) at 6 mg/kg and saline (n = 4) twice daily and once on weekends. After 8 weeks, rhesus macaques were infected with SHIVADE08. Using fecal samples, the 16s rDNA fecal microbiome was studied using the illumine sequencing platform. Similarly, flow cytometry was utilized to interrogate changes in myeloid modifications found in the brain, gut, and liver. Levels of SHIV DNA were estimated using the digital droplet polymerase chain reaction (dd-PCR) assay across different regions of gut and brain tissues. [file Image_1.TIF]

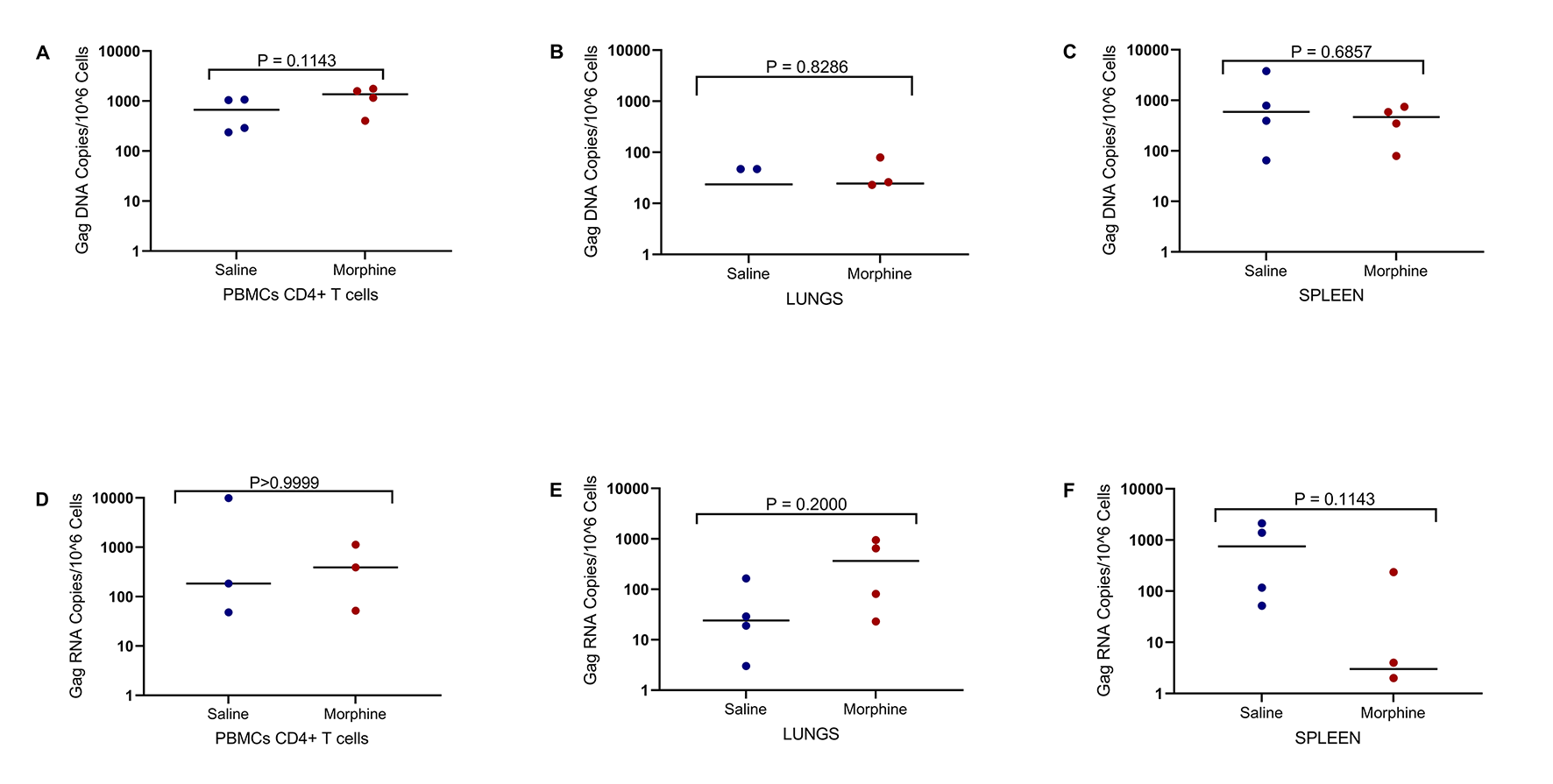

Supplement: Supplementary Figure 2 — Viral dynamics in peripheral blood and diverse tissues. Differences in Gag DNA levels found in (A) PBMC CD4+ T-cells (B) Lungs (C) Spleen in eight SHIV infected rhesus macaques exposed to either morphine or saline. Differences in Gag RNA levels found in (E) PBMC CD4+ T-cells (F) Lungs (G) Spleen in eight SHIV infected rhesus macaques exposed to either morphine or saline. [file Image_2.TIF]

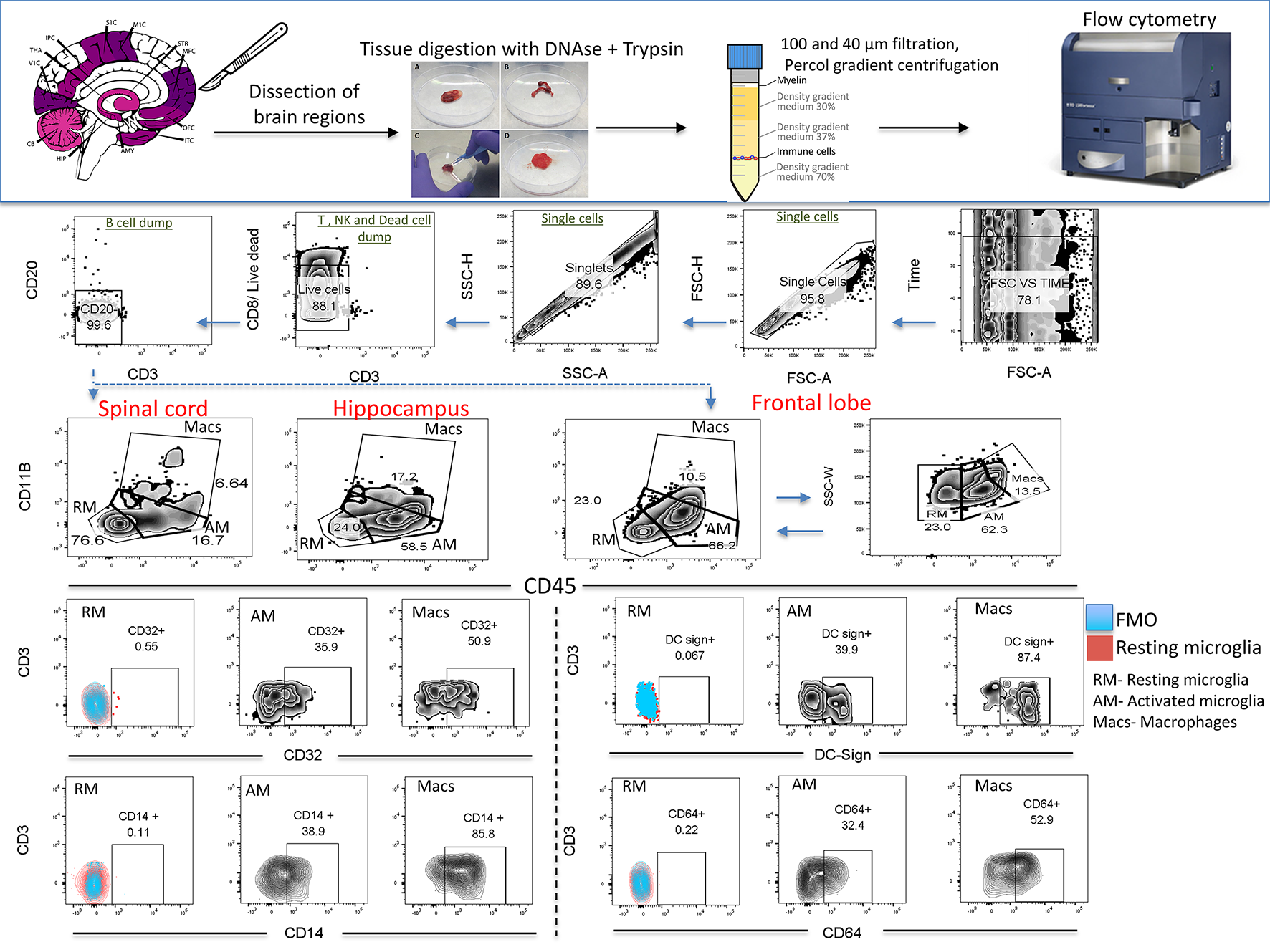

Supplement: Supplementary Figure 3 — Brain gating strategy used to interrogate brain myeloid (microglia and macrophages) found in diverse brain regions. Various brain regions were dissected and tissue digestion carried out using DNAse and Trypsin and obtained single-cell suspensions analyzed using the BD Fortesssax450. Detailed gating strategy involving an FSCA vs. Time to check the stability of the flow and obtain events only when an expected stream flows were obtained. Using Pulse Geometry scales comprised of FSC-H vs. FSC-A and SSC-H vs. SSC-A dot plots, we excluded doublets. Next, dump gates were used to eliminate unwanted populations. CD8α+ cells and Live Dead exclusion plus CD3+ T-cell removal guided the exclusion of CD3+ T-cells, NK cells, and dead cells. Next, the CD20 dot plots guided the removal of B-cells. Collectively, this ensured that the major populations studied in Brain cells were majorly myeloid cells. CD45 vs. CD11B plots were utilized to guide segregation of resting/activated microglia in addition to macrophages. Fluorescence minus one (FMO) controls were used to guide the proper placement of gates of diverse myeloid cell markers within the resting microglia, activated microglia and macrophages. [file Image_3.TIF]

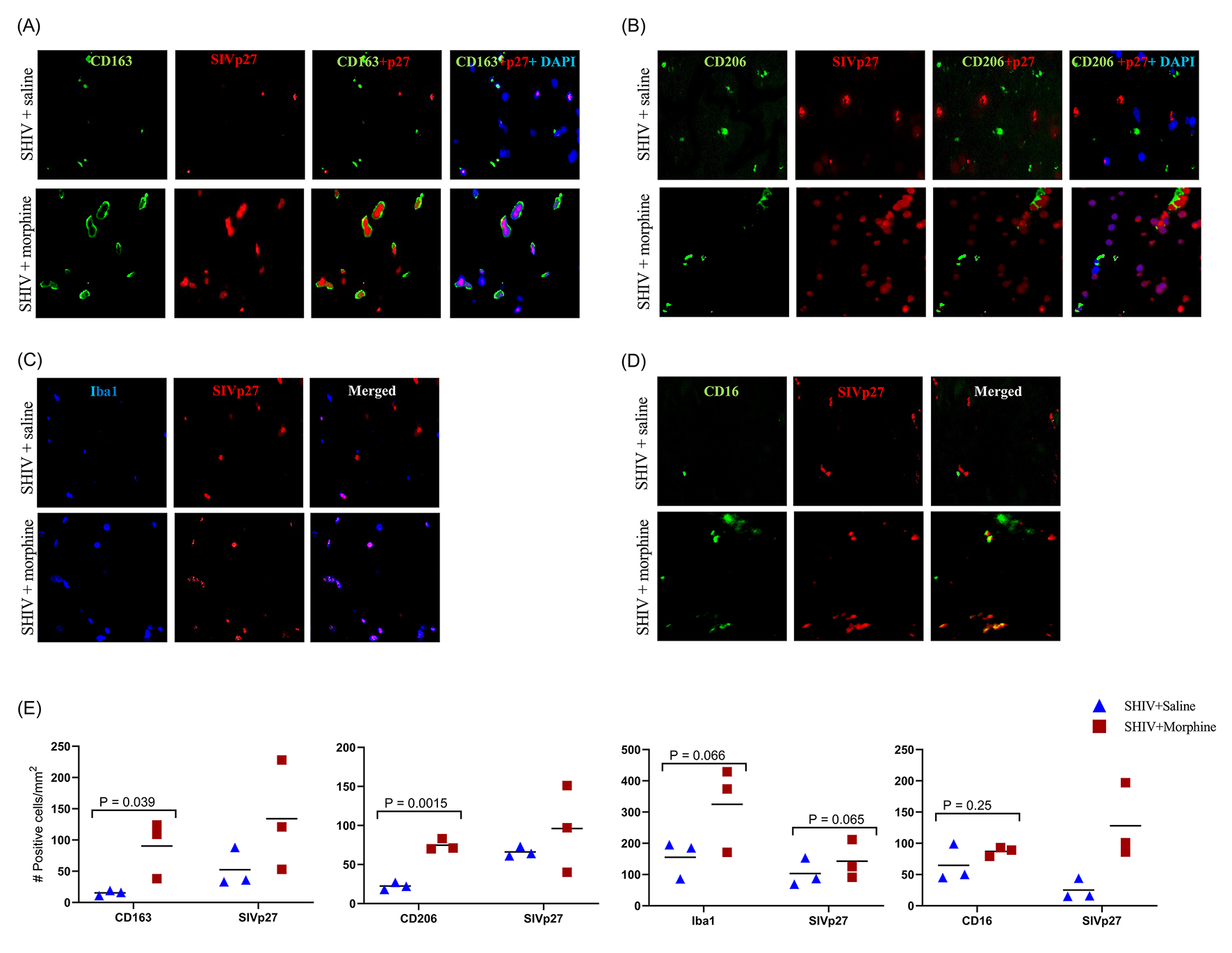

Supplement: Supplementary Figure 4 — Surface expression of diverse myeloid markers in the hippocampus of SHIV-infected saline or morphine-exposed rhesus macaques. Immunofluorescence images of the hippocampus was stained with different macrophage markers: (A) CD163 and (B) CD206 (green) together with SIV-p27 (red) and DAPI for nucleus was visualized by fluorescence microscope. (C) Single and combined staining for Iba 1 (blue), SIV-p27 (red) and merged co-expression of Iba 1and SIV-p27. (D) Single stained CD16, SIV p27 and CD16 (green) co-expressed with p27 in the brain of one representative rhesus macaque. The upper panel indicates SHIV + saline while panel (E) represents SHIV + morphine respectively. Quantitative analyses were done by two groups each n = 3 showing the number of cells per mm2 of tissue that stained positively for the markers of interest (p < 0.05 signifies statistical significance as carried out using unpaired T-tests). [file Image_4.TIF]
