# Supplementary material for: Systems biology analyses reveal enhanced chronic morphine distortion of gut-brain interrelationships in simian human immunodeficiency virus infected rhesus macaques
Source: Front Neurosci. 2022 Oct 13;16:1001544. doi: 10.3389/fnins.2022.1001544 (PMC9613112; doi:10.3389/fnins.2022.1001544)
Supplement: Supplementary file 5 [file Table_1.docx]

| **Animal ID** | **DOB** | **Gender** | **Age (years) at necropsy date** |  | **Group** | **Virus** | **Peak of SHIV Plasma Viremia (RNA copies/ml)** |
| --- | --- | --- | --- | --- | --- | --- | --- |
| 13L126 | 5/29/13 | Male | 6.2 |  | Morphine+SHIV | SHIV | 1.69E+07 |
| 14X046 | 7/15/14 | Male | 5 |  | Morphine+SHIV | SHIV | 9.87E+06 |
| 14X016 | 5/27/14 | Male | 5.1 |  | Saline+SHIV | SHIV | 7.64E+06 |
| 11N074 | 5/18/11 | Male | 8 .1 |  | Saline+SHIV | SHIV | 8.22E+06 |
| 12N015 | 4/19/12 | Male | 7.2 |  | Saline+SHIV | SHIV | 5.22E+06 |
| 12N044 | 5/17/12 | Male | 7.1 |  | Saline+SHIV | SHIV | 6.25E+05 |
| 11N097 | 6/7/11 | Male | 8 |  | Morphine+SHIV | SHIV | 3.38E+06 |
| 12N060 | 5/29/12 | Male | 7.10 |  | Morphine+SHIV | SHIV | 2.89E+07 |
|  |  |  |  |  |  |  |  |

**Supplementary Table 1:** Characheristics of Rhesus Macaques that were included for the study that comprised of allocated animal IDs, Age, Sex and Peak Viremia.
