# Supplementary material for: Systems biology analyses reveal enhanced chronic morphine distortion of gut-brain interrelationships in simian human immunodeficiency virus infected rhesus macaques
Source: Front Neurosci. 2022 Oct 13;16:1001544. doi: 10.3389/fnins.2022.1001544 (PMC9613112; doi:10.3389/fnins.2022.1001544)
Supplement: Supplementary file 6 [file Table_2.docx]

**Supplementary Table 2:** (A) Comprehensive list of antibodies used for flow cytometry analysis of brain/ gut myeloid cell phenotypes within diverse regions. (B) Details of soluble analytes (biomarkers) included in the 16 plex Luminex panel used for our study.

1. **List of Antibodies Used in 18 color flow cytometry Panel (list).**

| **Epitope** | **Conjugate** | **Clone** | **Supplier** | **Catalogue** | **Dilution** |
| --- | --- | --- | --- | --- | --- |
| CD32 | APC | FUN-2 | Biolegend | 303208 | 1:50 |
| CD45 | FITC | D058-1283 | BD | 557803 | 1:40 |
| CX3CR1 | PE | K0124E1 | Biolegend | 355704 | 1:40 |
| HLA-DR | PE Texas Red | MHLDR17 | Life Tech | 2086613 | 1:30 |
| CD64 | APC-eFlour 780 | 10.1 | Invitrogen | 47064942 | 1:30 |
| *CD11C | BUV395 | B-ly6 | BD Horizon | 563787 | 1:20 |
| Dc Sign | PE Cy5 | DCN46 | BD Pharm | 558263 | 1:40 |
| CD163 | BV786 | GHI/61 | BD OptiBuild | 741003 | 1:40 |
| CD3 | AF700 | SP34-2 | BD Pharm | 557917 | 1:50 |
| CD16 | Pacific Blue | 3G8 | BD Pharm | 558122 | 1:50 |
| CD206 | BV650 | 19.2 | BD OptiBuild | 740598 | 1:50 |
| CD8α | BV510 | SK1 | Biolegend | 344732 | 1:50 |
| Live/Dead | Aqua | N/a | Biolegend | 423102 | 1:1000 |
| CD11B | PE Cy7 | M1/70 | Biolegend | 101216 | 1:50 |
| CD14 | BV570 | M5E2 | Biolegend | 301832 | 1:50 |
| CD20 | BUV805 | 2H7 | BD Biosciences | 612905 | 1:80 |

Additional reagents: Invitrogen Anti-Fc Receptor Binding Inhibitor Polyclonal, Bioscience™ supplied by Life Technologies (Ref:14-9161-73). *CD11C clone was included in this panel, it was not cross reactive and stained poorly in brain/gut cells and was not utilized for analyses.

**(B) List of Soluble Analytes (Biomarkers) used for Luminex Panel**

| **Product name** | **Supplier** | **Catalogue Number** |
| --- | --- | --- |
| MIP-1a | Invitrogen/ProcartaPlex | EPX01A-42029-901 |
| MPO | Invitrogen/ProcartaPlex | EPX01A-12038-901 |
| LYVE-1 | Invitrogen/ProcartaPlex | EPX010-12239-901 |
| IL-8 | Invitrogen/ProcartaPlex | EPX01A-10204-901 |
| Eotaxin | Invitrogen/ProcartaPlex | EPX01B-42120-901 |
| IDO | Invitrogen/ProcartaPlex | EPX01A-12213-901 |
| I-TAC | Invitrogen/ProcartaPlex | EPX01A-42124-901 |
| MIG | Invitrogen/ProcartaPlex | EPX01A-40285-901 |
| MIF | Invitrogen/ProcartaPlex | EPX01A-42127-901 |
| MIP-1b | BioRad | 171B5023M |
| RANTES | BioRad | 171B5025M |
| IL-1Ra | R&D Systems | LUHM280 |
| CRP | R&D Systems | LOBM1707 |
