# Supplementary material for: Systems biology analyses reveal enhanced chronic morphine distortion of gut-brain interrelationships in simian human immunodeficiency virus infected rhesus macaques
Source: Front Neurosci. 2022 Oct 13;16:1001544. doi: 10.3389/fnins.2022.1001544 (PMC9613112; doi:10.3389/fnins.2022.1001544)
Supplement: Supplementary file 7 [file Table_3.docx]

**Supplementary Table 3:** Probe/ Primer design and description used for estimates of SHIV DNA using Digital Droplet Polymerase Chain Reaction (Dd PCR):

**Cycling conditions:**

| Cycling step | Temp (C) | Time | Number of cycles |
| --- | --- | --- | --- |
| Enzyme activation | 95 | 10 min | 1 |
| Denaturation | 94 | 30 sec | 40 |
| Annealing/extension | 60 | 1 min |  |
| Enzyme deactivation | 98 | 10 min | 1 |
| Hold | 4 | Infinite | 1 |
| Use a heated lid set to 105°C and set the sample volume to 40uL | | | |
| Probe/ Primer design  SIVgag Probe:  5’-/56-FAM/ CTT CCT CAG /ZEN/ TGT GTT TCA CTT TCT CTT CTG CG /3IABkFQ/-3’  5¢-(FAM) CTT CPT CAG /ZEN/ TKT GTT TCA CTT TCT CTT CTG CG-(BHQTM1)-3¢,  5' 6-FAM (Fluorescein):  FAM is the most commonly used fluorescent dye attachment  Code = /56-FAM/  ZEN  A traditional probe is 20−30 bases in length with a terminal dye and quencher. The internal ZEN quencher thus shortens the distance between dye and quencher, and in combination with the terminal 3’ quencher, provides a higher degree of quenching and lowers initial background. This quencher is placed internally between the 9th and 10th base from the reporter dye on the 5’ end of a probe sequence and 20-30bp before the 3’ quencher.  Code = /ZEN/  3' Iowa Black® FQ  Iowa Black® FQ has a broad absorbance spectrum ranging from 420 to 620 nm with peak absorbance at 531 nm. This quencher is ideal for use with fluorescein and other fluorescent dyes that emit in the green to pink spectral range.  Code = /3IABkFQ/  **Primers:**  Fwd: GTCTGCGTCATCTGGTGCATTC Rev: CACTAGGTGTCTCTGCACTATCTGTTTTG | | | |
